# Evolution of High-Temperature Oxygen Clusters and Radical Release: A Molecular Dynamics Study in Pure Oxygen and Titanium Tetrachloride Oxidation Environments

**DOI:** 10.3390/ma19061048

**Published:** 2026-03-10

**Authors:** Dongqin Li, Jie Zhou, Ping Lu, Linfei Li, Zhuo Sheng, Yunmin Chen, Rong Yu, Caiqing Wang, Xiumin Chen, Dachun Liu

**Affiliations:** 1 State Key Laboratory of Complex Nonferrous Metal Resources Clean Utilization, School of Metallurgical and Energy Engineering, Kunming University of Science and Technology, Kunming 650093, China15554136867@163.com (C.W.); 2 State Key Laboratory of Vanadium and Titanium Comprehensive Utilization, Pangang Group Research Institute Co., Ltd., Panzhihua 617000, China; 3 Beijing DP Technology Co., Ltd., Beijing 100089, China; 4 National Engineering Research Center of Vacuum Metallurgy, Kunming University of Science and Technology, Kunming 650093, China

**Keywords:** high-temperature, oxygen clusters, free radical, molecular dynamics

## Abstract

**Highlights:**

**What are the main findings?**

- The “oxygen clustering–radical release” pathway is identified in high-temperature oxidation.
- Odd-numbered oxygen clusters (dominated by O_3_) are reactive while even-numbered are inert.
- Reaching a critical size, the cluster releases radicals that persist in pure O_2_ and TiCl_4_-containing environments.

**What are the implications of the main findings?**

- Provides a novel mechanism for the controlled synthesis of functional oxide materials.
- Offers a new perspective for understanding the high-temperature oxidation corrosion mechanisms of alloy materials.
- Holds implications for the high-temperature combustion processes of fuel materials.

**Abstract:**

Despite decades of research into high-temperature oxidation kinetics, the atomic-scale origin of oxygen radical formation—a critical driver of the synthesis of functional oxide materials, oxidation corrosion of alloys, and combustion of fuels—remains elusive. Here, we unveil a previously unrecognized “oxygen clustering–radical release” pathway at the molecular level, wherein transient van der Waals aggregates of oxygen molecules, rather than isolated O_2_, serve as the primary radical source. Through an integrated computational strategy combining first-principles calculations and deep neural network potential function molecular dynamics, we demonstrate that oxygen clusters exhibit a pronounced odd–even oscillation in stability: even-numbered clusters (especially O_6_/O_8_ subunits) are chemically inert, whereas odd-numbered clusters (dominated by O_3_) possess high reactivity. Kinetically, clusters evolve via reversible aggregation–dissociation, and upon reaching a critical size, undergo structural collapse accompanied by radical release—a process initiated predominantly by large odd-numbered clusters. This mechanism persists in both pure O_2_ and TiCl_4_-containing environments, with TiCl_4_ modulating only the aggregation kinetics, not the fundamental pathway. Our work establishes oxygen clustering as a universal, spontaneous source of radicals in high-temperature oxidation, providing a new molecular-level mechanistic framework for understanding and controlling radical-driven processes in materials synthesis, corrosion and combustion.

## 1. Introduction

The controlled synthesis of functional oxide materials, alongside the mitigation of high-temperature corrosion and optimization of combustion, is closely related to a fundamental understanding of oxygen’s behavior under extreme conditions. In the high-temperature oxidation of titanium-based materials, oxygen (O_2_) participates in complex physicochemical processes to form titanium dioxide (TiO_2_), a key functional oxide with a broad spectrum of applications in coatings, photocatalysis, and solar cells. Although extensive studies have explored the kinetics and thermodynamics of oxygen in macroscopic high-temperature reactions [1,2,3], most models rest on a core assumption: oxygen consistently exists as isolated O_2_ dimers [4,5,6]. This simplification may overlook the potential formation of intermolecular associations and transient polymers in oxygen under high-temperature, high-pressure conditions, along with their potential influence on the nucleation and growth of TiO_2_ nanostructures. Despite some experimental hints of such clustered species, their role has been largely absent from mainstream kinetic process.

As early as the 1950s, Grundland [7] first observed an ion current with a mass number of 64 when oxygen passed through an ozone generator, suggesting the possible existence of O_4_ molecules. With advancements in experimental techniques, particularly mass spectrometry and infrared spectroscopy, the existence of oxygen dimers (O_2_-O_2_) was gradually confirmed [8]. The assembly of oxygen clusters is governed by weak intermolecular forces, as the binding energy between O_2_ molecules is significantly lower than that characteristic of conventional chemical bonds. O_4_ is considered a “bound dimer” rather than a transient collision pair. Under cryogenic conditions, where oxygen exists in solid form, the geometric structures, electronic properties, and magnetic evolution of (O_2_)_n_ clusters have been widely studied [9,10,11]. Research by Santoro [12] indicates that oxygen molecules within condensed phases tend to aggregate into clusters with long-range order. Notably, in solid oxygen, (O_2_)_4_ tetramers are formed through weak O_2_-O_2_ chemical interactions when subjected to pressures above 10 GPa. Based on high-accuracy quantum chemical calculations, Gadzhiev [13] computed the structures, energies, and vibrational frequencies of O*_n_* (*n* = 1–6) to elucidate the properties of products formed during solid O_2_ irradiation. Forte [14] predicted the free-space structures of O_6_, O_8_, and O_12_. Besides neutral clusters, oxygen is capable of forming charged cluster ions (e.g., O_4_^+^ and O_4_^−^). The stability differences among cluster ions stem from charge distribution and polarization effects. O_4_^−^ exhibits higher bond energy than O_4_^+^ due to enhanced polarization from the extra electron. Conway [15,16,17,18] conducted an investigation of O_4_^+^ to O_18_^+^ ion clusters under conditions of low temperature and low pressure, determining their thermodynamic parameters. The study also identified the presence of “magic number” phenomena within the cluster size distribution at reduced temperatures.

Advancements in computational chemistry and characterization methodologies have enabled researchers to attain a more comprehensive understanding of the equilibrium properties and mechanistic pathways of oxygen radical reactions. Li [19] investigated the combustion characteristics of biogas under oxygen-enriched conditions, noting that O radicals are generated during combustion. Elevated oxygen concentrations facilitate the generation of H, O, and OH radicals, thereby accelerating the kinetics of exothermic reactions. Yamamoto [20] investigated the dynamic behavior of oxygen radicals within microwave plasma. In this context, high-energy electron collisions induce the dissociation of O_2_ molecules, generating O radicals. These radicals subsequently react with hydrocarbon fuels or hydrogen radicals to produce hydroxyl (OH) species, thereby facilitating the acceleration of the oxidation process. Sun [21] demonstrated that, under conditions characterized by elevated temperature and energy, such as those present in plasma-assisted combustion, molecular oxygen (O_2_) can undergo direct dissociation to produce oxygen radicals. Song [22] proposed that the interconversion processes among H, O and OH radicals is the principal origin of oxygen radicals in combustion systems, emphasizing that the surface activation mechanism of O radicals plays a critical role in catalytic combustion. Zhang [23], studying the combustion characteristics of the zero-carbon renewable fuel ammonia, found that oxygen radicals, specifically O and OH, are critical in facilitating NH_3_ flame propagation by significantly enhancing reaction rates within the reaction zone. Chen et al. [24] proposed a radical cycle model for coal spontaneous combustion, demonstrating that initial reactions accumulate heat and promote the generation of active peroxyl radicals (R-O-O∙) and hydroxyl radicals (OH∙).

The studies referenced above demonstrate that, under cryogenic and condensed-phase conditions, oxygen molecules tend to aggregate into clusters predominantly composed of either oxygen molecules or ions. Conversely, in high-temperature combustion environments, oxygen primarily participates in reactions via dissociation into radicals. Bazarkina et al. [25] used in situ X-ray spectroscopy and electronic structure calculations to reveal the formation of uranium cubic octahedral clusters during UO_2_ oxidation. This study confirms that cluster formation can occur in high-temperature oxidation processes. Nonetheless, there is a paucity of research investigating whether O_2_ continues to form cluster structures similar to those observed in low-temperature environments when subjected to high-temperature conditions, as well as how the possible presence of such clusters may affect the production of oxygen radicals and the associated reaction kinetics. This gap arises partly from the substantial challenges associated with experimentally observing and theoretically modeling oxygen behavior under extreme high-temperature conditions, and partly from the inherent limitations of conventional research methodologies when applied to complex systems at elevated temperatures. In recent years, with the rapid development of artificial intelligence and high-performance computing methods, reaction mechanism research driven by first-principles calculation and deep learning is gradually maturing. Liu [26] employed Density Functional Theory (DFT) and AIMD to study the reaction pathways, thermodynamics, and kinetics of organic chlorine conversion to HCl during coal combustion. Ding [27] combined Deep Potential Molecular Dynamics (DPMD) and Ab Initio Molecular Dynamics (AIMD) simulations to study the interfacial structure and dynamics of amorphous titanium dioxide (a-TiO_2_) in aqueous coating systems. Liu [28] utilized AIMD to theoretically investigate the thermal decomposition processes and primary reaction pathways of aqueous solutions of Hydroxylammonium Nitrate (HAN) as well as HAN mixtures with fuels such as methanol and ethyl ammonium nitrate. Qing [29] used LAMMPS simulations to study the combustion process of CH_4_ and O_2_ on a Pd catalyst surface within a spiral microchannel. The study involved calculating atomic-level behavior and thermodynamic parameters under varying initial temperatures and oxygen concentrations. Liu [30] integrated first-principles data to train neural network potential functions and employed the DPMD method to simulate the growth of SiC nanowire and track the aggregation behavior of CO/SiC molecules on a graphite substrate.

This study employs a methodology integrating first-principles calculations with deep neural network potential function molecular dynamics simulations to systematically investigate the dynamic evolution of oxygen clusters during high-temperature reaction. It reveals their microscopic formation mechanisms at the atomic scale, providing theoretical foundations and simulation frameworks for understanding oxygen behavior in high-temperature oxidation reactions.

## 2. Computational Methods

### 2.1. Cluster Configuration Calculations

The initial geometries of oxygen clusters O*_n_* (*n* = 2–25) with diverse geometric features from the trajectories of DPMD (Deep Potential Molecular Dynamics) simulations at different temperatures were constructed using the Materials Studio (MS) 2017 [31]. To identify the most stable ground-state structures, geometry optimizations were performed using the Gaussian 16W (Revision A.03) software package at the M06L/6-311++G(d,p) level of theory [32], with convergence criteria of 1.0 × 10^−6^ Ha for energy, 0.00045 Ha/Å for force, and 0.0018 Å for displacement.

For each optimized structure, to accurately determine its ground spin state, we systematically calculated the energies for different multiplicities at the M06L/6-311++G(d,p) level. The ground state was identified as the spin configuration with the lowest total energy (E_min), based on the quantum mechanical variational principle. Subsequently, frequency calculations were performed on the optimized structures at the same level to confirm the absence of imaginary frequencies.

To systematically analyze the structures of oxygen clusters, we defined the following criteria. A distance threshold of 3.04 Å for O-O interatomic distances was set to identify atoms belonging to the same molecular subunit. This value is equal to the sum of the van der Waals radii of oxygen atoms [33], ensuring that we capture intermolecular interactions within a subunit. To determine whether two subunits form a larger cluster, we adopted the criterion that if the distance between any pair of oxygen atoms (O–O) from different subunits does not exceed 3.3 Å, the two subunits are considered connected and are classified as part of the same cluster.

The electrostatic potential (ESP) maps were generated and rendered using GaussView 6.0. Molecular orbital diagrams, including the highest occupied molecular orbital (HOMO) and the lowest unoccupied molecular orbital (LUMO), were visualized using Visual Molecular Dynamics (VMD 1.9.1) software. The energy of the HOMO-LUMO gap was computed based on the orbital energies derived from the Gaussian single-point calculation.

### 2.2. Molecular Dynamics Simulations

Molecular dynamics (MD) simulations were performed using the LAMMPS (2 Aug 2023 version) package [34] with the deep neutral network potential model that trained by ourselves. The detailed training process and validation of this potential model can be found in our previous work, which is presented in the Supplementary Materials [35,36,37,38]. The initial simulation cells for both the pure oxygen system and the ternary Ti-O-Cl system were constructed using the Amorphous Cell module in Materials Studio, ensuring random molecular distribution without unrealistic atomic overlaps. The systems were defined as follows: The oxygen system consisted of 406 O_2_ molecules and 12 O atoms, modeling a high-temperature oxygen-rich environment with a minor radical component. The Ti-O-Cl system contained 2624 O_2_ molecules and 1914 TiCl_4_ molecules, representing a stoichiometric mixture for the oxidation reaction.

All simulations were conducted in the isothermal-isobaric (NPT) ensemble. The temperature was maintained at the target reaction condition of 1573 K using a Nosé-Hoover thermostat [39]. The pressure was controlled independently at 3 atm and 6 atm using a Nosé-Hoover barostat to investigate pressure effects. The simulation was performed for 10 ns and 8 ns on the oxygen and Ti-O-Cl systems, respectively, with a time step of 0.2 fs.

## 3. Results and Discussion

### 3.1. The Ground-State Structures of O_n_ (n = 2–25) Clusters

**Figure 1:**
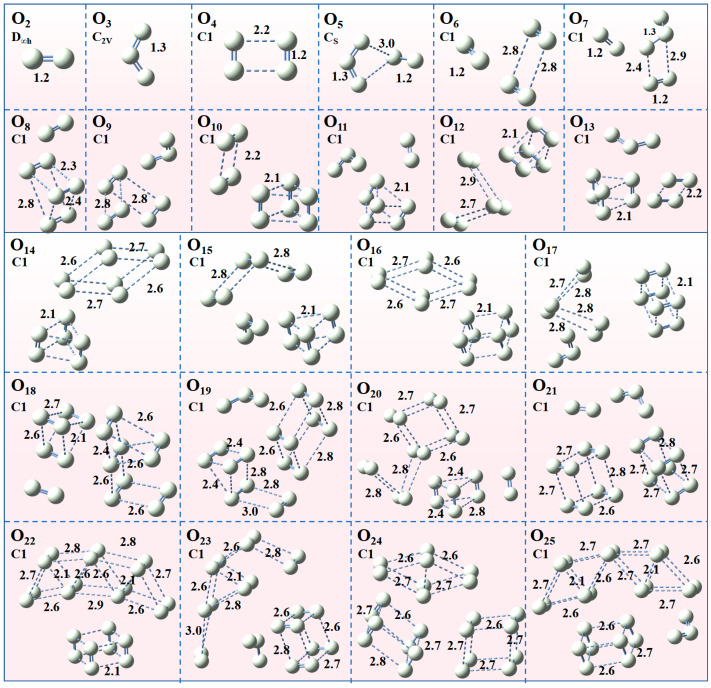
Structural diagram of the ground-state cluster. Symmetry labels (C1, C_s_, C_2v_, D_∞h_) denote point group symmetries. Numbers (e.g., 1.2, 2.2, 2.8) indicate interatomic bond lengths in Å.

Figure 1 illustrates the ground-state structures of O*_n_* (*n* = 2–25) clusters, revealing their structural evolution patterns based on the assembly of fundamental subunits. Analytical results demonstrate that all clusters with even numbers are constituted by n/2 O_2_ subunits, whereas clusters with odd numbers comprise a single O_3_ subunit in conjunction with several O_2_ subunits. In smaller clusters (*n* = 2–5), the structures preserve a degree of symmetry. Conversely, for clusters with *n* ≥ 6, the symmetry is diminished, resulting in all clusters belonging to the C1 point group. The O-O bond lengths in small clusters (O_2_, O_3_), measuring 1.21 Å and 1.30 Å respectively, characterize their stable covalent bonding characteristics. For *n* ≥ 4, the cluster structures transform into aggregates of O_2_ and O_3_ subunits through weak interactions. Specifically, O_4_ and O_5_ are constituted by (O_2_)_2_ and O_2_·O_3_ respectively. The inter-subunit distances increase from 2.2 Å to 3.04 Å, indicating a reduction in interaction strength. For larger clusters with *n* > 5, their structures are formed through the cooperative assembly of various smaller subunits (e.g., O_2_, O_3_, O_4_, O_6_, O_8_). The O_6_ cluster consists of three O_2_ subunits, with a distance of 2.8 Å between two O_2_ subunits (equivalent to an O_4_ subunit), slightly longer than the corresponding bond length within an independent O_4_ cluster. Analysis of the dihedral angles presented in Table S1 of Supplement S2 reveals that in isolated O_4_ clusters, the characteristic dihedral angle approaches 180°, indicating a nearly ideal planar quadrilateral configuration with negligible torsional strain. However, when O_4_ is embedded as a subunit in the O_6_ cluster, its dihedral angle becomes 174.4°, showing a 5.6° distortion compared to the isolated O_4_ cluster. Other critical dihedral angles within the O_6_ cluster exhibit distortions approaching 10°, suggesting that the cluster exists in a relatively unstable high-energy state. The O_8_ cluster is formed by the combination of O_2_ and O_6_ subunits, with a dihedral angle of 177.0° at the junction, indicating slight distortion. The configuration of the O_6_ subunit exhibits notable differences compared to that of an isolated O_6_ cluster. Specifically, in the isolated cluster, the O_2_ subunits are positioned at greater intervals, whereas within the O_6_ subunit, the separation between each pair of O_2_ subunits is constrained to approximately 2.3–2.4 Å, accompanied by internal dihedral angles nearing 180°. The findings suggest that within the restricted environment of the O_8_ cluster, the O_6_ subunit displays intensified O-O interactions, resulting in markedly greater stability relative to the isolated O_6_ cluster. Furthermore, in larger clusters ranging from O_10_ to O_15_, the presence of similarly robustly bound O_6_ subunits is consistently evident. As the cluster size increases beyond *n* = 15, discernible cube-like or rhombus-like O_8_ subunits begin to emerge. In O_16_ and O_17_ clusters, the O_2_ subunit spacing within the O_8_ subunits shortens to 2.1 Å, with critical internal dihedral angles highly approaching 180° (≥179.6°). This strongly suggests that both cube-like and rhombus-like O_8_ subunits can form highly stable configurations with minimal torsional strain in large clusters. However, upon reaching a size of *n* ≥ 19, the O_2_-O_2_ bond lengths within the O_8_ subunits predominantly elongate to a range of 2.6–2.8 Å, suggesting a decline in structural stability with the further increase in cluster size.

Notably, a newly emerging O_14_ subunit was observed in larger clusters such as O_22_ and O_25_. The O_22_ cluster consists of O_14_ and O_8_ subunits, while the O_25_ cluster further incorporates an O_3_ subunit, forming an O_14_-O_8_-O_3_ combination. The O-O distance within the O_14_ subunit of the O_25_ cluster increases relative to that in O_22_, which indicates that the incorporation of an O_3_ unit reduces the structural compactness of adjacent even-atom subunits. Similarly, in the O_23_ cluster (comprising O_12_-O_8_-O_3_), the O-O bond on the O_3_-adjacent side of the O_12_ subunit elongates to 3.0 Å, significantly longer than typical values. This pronounced elongation suggests a region of marked instability that may function as an active site for cluster dissociation.

The structural analysis identifies distinct patterns in interatomic distances within the cluster. Specifically, the oxygen-oxygen bonds within O_2_ or O_3_ molecules exhibit short covalent bond lengths of approximately 1.2 to 1.3 Å, as shown by the solid line in Figure 1. The distances between O_2_ molecules within the subunit vary from 2.1 to 3.04 Å as indicated by the dashed line in Figure 1, which are smaller than the combined van der Waals radii (3.04 Å). In contrast, the distances between subunits are observed to be greater than 3.04 Å (unlabeled in the figure for clarity). This clearly reflects the synergistic interaction between strong covalent bonds within subunits and weaker electrostatic or van der Waals forces between subunits. Furthermore, the emergence and distribution of characteristic subunit structures within oxygen clusters exhibit pronounced size dependence: O_4_ subunits predominantly exist in small clusters with *n* < 10; O_6_ subunits are common in medium-sized clusters with *n* = 8–20, while O_8_ subunits consistently remain stable within large clusters with *n* ≥ 14. When O_6_ or O_8_ subunits are embedded within larger clusters as structural units, their stability significantly surpasses that of their independent cluster states. In contrast, other subunits unable to form such stable O_6_ or O_8_ configurations generally exhibit structural relaxation features such as increased bond lengths and distorted dihedral angles. The variation in structural stability observed at the subunit level constitutes a fundamental structural factor underlying the pronounced disparities in the overall stability of oxygen clusters of varying sizes.

### 3.2. Energetic Stability in the Clusters

The thermodynamic stability of atomic or molecular clusters can be quantitatively assessed through parameters such as the average binding energy (E_b_) and the second-order differential energy (Δ_2_E). Furthermore, their electronic structural stability is closely related to the HOMO-LUMO gap (ΔE_H-L_) and the dipole moment.

For oxygen atom clusters, the average binding energy and second-order differential energy can be calculated using the following equations [40,41]:

E_b_ = (*n*∙E(O) − E(O*_n_*))/*n*

Δ_2_E(O*_n_*) = E(O*_n_*_+1_) + E(O*_n_*_−1_) − _2_E(O*_n_*)

where E(O): Energy of a single oxygen atom; E(O*_n_*): Total energy of a cluster composed of *n* oxygen atoms; *n*: Number of atoms in the cluster; E(O*_n_*_+1_) and E(O*_n_*_−1_) are the energies of the adjacent clusters, respectively.

**Figure 2:**
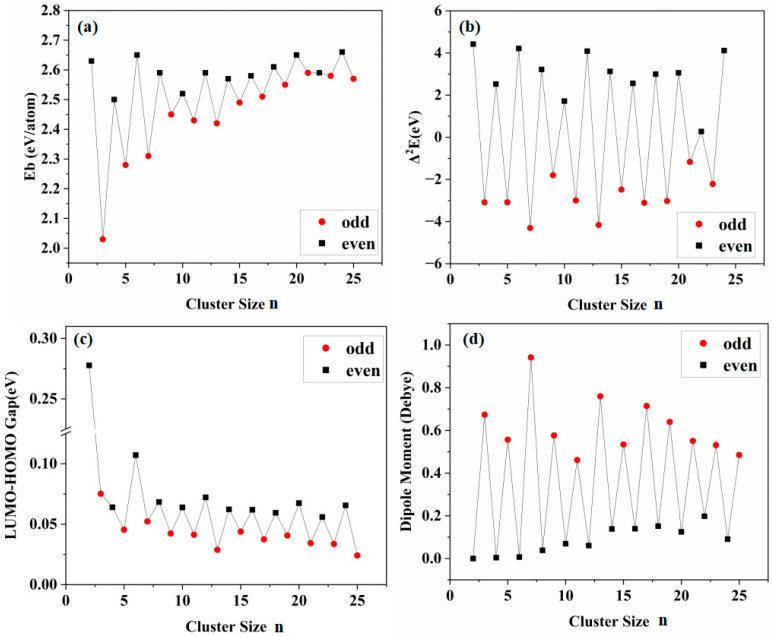
Evolution of thermodynamic properties with cluster size *n*. (**a**) Average binding energy (E_b_). (**b**) Second-order difference energy (Δ^2^E). (**c**) HOMO-LUMO gap. (**d**) Dipole moment. (odd: *n* = 3, 5, 7…25; envn: *n* = 2, 4, 6…24).

Figure 2a shows the variation in the average binding energy (E_b_) of O*_n_* (*n* = 2–25) clusters with increasing cluster size. The analysis reveals a pronounced odd–even oscillation in the binding energy E_b_ with respect to cluster size: The binding energy (E_b_) values of clusters with even numbers of atoms are typically greater than those of their neighboring odd-atom clusters, suggesting that even-atom clusters exhibit increased thermodynamic stability. Further analysis reveals size-dependent variations in the binding energies of clusters with odd and even numbers of atoms. The binding energies of clusters with even numbers of atoms display minor fluctuations within a limited range, with variations not exceeding 0.15 eV per atom, indicating that their stability is largely independent of cluster size. Conversely, the binding energies of clusters containing an odd number of atoms demonstrate a marked positive correlation with size. This trend is especially evident in the size range of *n* = 3 to 9, where binding energies increase rapidly, subsequently followed by a more gradual reduction in the rate of growth. The maximum energy difference reaches 0.56 eV/atom, demonstrating that larger odd-atom clusters possess significantly enhanced thermodynamic stability compared to their smaller counterparts. This phenomenon not only confirms the inherent size dependence of odd-atom clusters but also validates the results from ground-state structural analysis: high-stability subunits such as O_6_ and O_8_ provide crucial structural frameworks for larger clusters (*n* > 8), which fundamentally contribute to their overall stability enhancement. Additionally, the binding energy of O_3_ represents the lowest value among all clusters and is significantly lower than that of O_2_. This computational result aligns remarkably well with established experimental observations: ozone (O_3_) spontaneously undergoes disproportionation to form oxygen (O_2_) under ambient conditions, whereas the conversion of O_2_ to ozone requires external energy input (e.g., through electrical discharge), thereby energetically confirming the thermodynamic instability of O_3_.

As shown in Figure 2b, the second-order energy difference (Δ_2_E) serves as a crucial indicator for characterizing size-dependent cluster stability: when Δ_2_E > 0, it indicates that the cluster of that particular size exhibits higher stability compared to its neighboring sizes, whereas Δ_2_E < 0 suggests lower stability. The analysis demonstrates that Δ_2_E exhibits significant odd–even oscillatory behavior with respect to cluster size *n*: all even-atom clusters display positive Δ_2_E values, while odd-atom clusters consistently show negative Δ_2_E values. This pattern clearly demonstrates that even-atom oxygen clusters possess superior thermodynamic stability compared to their odd-atom counterparts.

The HOMO-LUMO energy gap serves as a key parameter characterizing the stability of a system’s electronic structure and its chemical inertness. As shown in Figure 2c, O_2_ exhibits the largest energy gap, indicating the highest kinetic stability and chemical inertness. This aligns with oxygen’s most stable natural existence as a diatomic molecule. The evolution of the energy gap with cluster size exhibits highly correlated even–odd oscillations with thermodynamic parameters (E_b_, Δ^2^E): the energy gap of even-atom clusters show a systematic positive shift, averaging approximately 0.025 eV higher than odd-atom clusters. This further confirms, at the electronic structure level, that even-sized clusters possess higher chemical stability. Conversely, odd-atom clusters not only exhibit lower HOMO-LUMO gaps but also show a decreasing trend with increasing size (especially beyond *n* > 19), indicating that larger odd clusters possess less stable electronic structures and higher chemical reactivity.

Dipole moment results, as a key indicator of charge distribution symmetry, further corroborate the parity difference in cluster stability: all even clusters (especially O_2_, O_4_, O_6_, O_8_) exhibit dipole moments approaching zero, confirming their symmetric charge distribution—the very electronic foundation of even clusters’ high stability. Conversely, the significantly increased dipole moments of odd-atom clusters indicate asymmetric charge distribution and deviation from the positive and negative charge centers. At the electronic structure level, this is intrinsically linked to their lower thermodynamic stability.

Based on a comprehensive analysis of binding energy, second-order difference energy, HOMO-LUMO gap, and dipole moment, both O_6_ and O_8_ are identified as highly stable fundamental structural units. This conclusion is highly consistent with the distribution pattern observed in oxygen clusters: when *n* ≥ 10 and *n* ≥ 14, the structurally highly stable O_6_ and O_8_ subunits begin to exert their dominance, emerging as the defining features of the large-sized clusters. Furthermore, the overall stability of the O_n_ clusters does not vary monotonically with cluster size but is primarily governed by the parity (even/odd nature) of the number of oxygen atoms, demonstrating significant oscillatory behavior. Even-atom oxygen clusters exhibit superior thermodynamic stability and chemical inerticity from both thermodynamic and electronic structure perspectives. In contrast, while the thermodynamic stability of odd-atom clusters increases with size, their chemically activity, which is governed by electronic structure, decreases. This pattern indicates that as the size increases, the influences of geometric structure and electronic structure on the stability of odd-atom clusters engage in a competitive trade-off relationship, leading to a fundamental shift in the underlying stability mechanism.

### 3.3. Electrostatic Potential and Orbital Analysis

Based on the aforementioned analysis of binding energy, second-order difference energy, HOMO-LUMO gap, and dipole moment, it is collectively demonstrated that the stability of O_n_ clusters is closely related to their electronic structure and charge distribution. To intuitively reveal the underlying microscopic mechanisms, the Electrostatic Potential (ESP) and Frontier Molecular Orbitals (FMO) of the oxygen clusters will be subjected to visual analysis and discussion.

**Figure 3:**
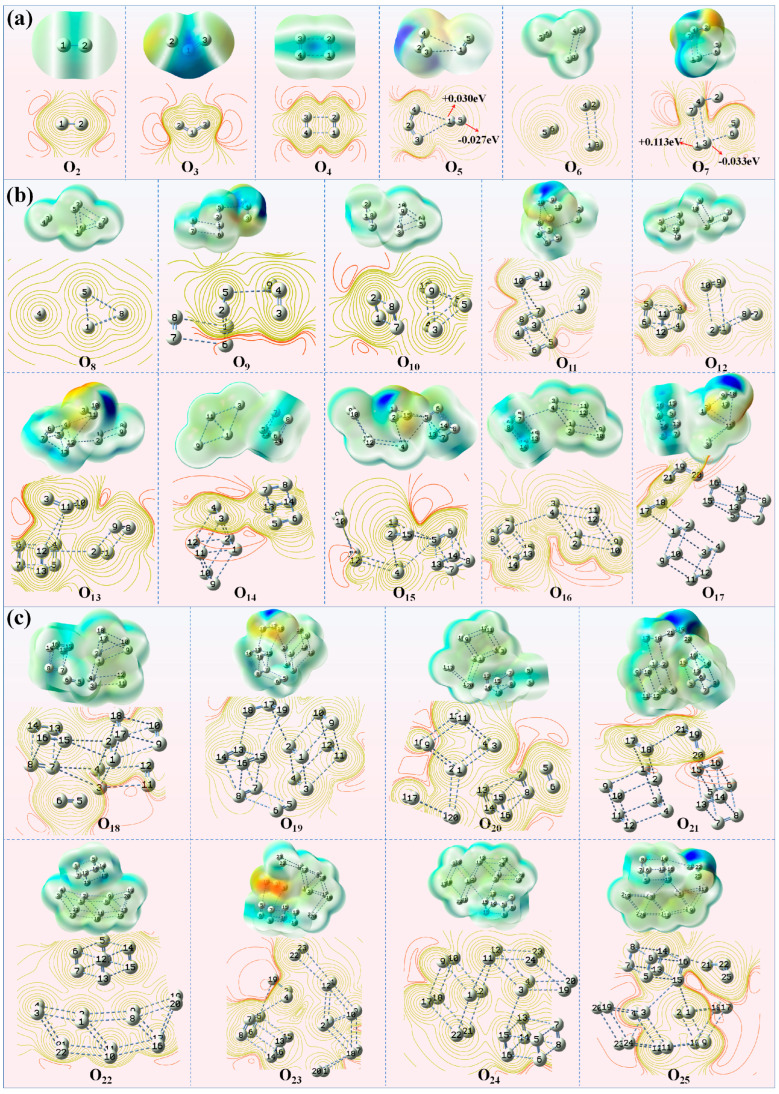
Electrostatic Potential (ESP) of the clusters (**a**) *n* = 2–7 (Red arrows indicate atomic charges); (**b**) *n* = 8–17; (**c**) *n* = 18–25. Colors of ESP represent values (in eV) from −2.5 × 10^−2^ (red, electron-rich) to +2.5 × 10^−2^ (blue, electron-poor).

**Figure 4:**
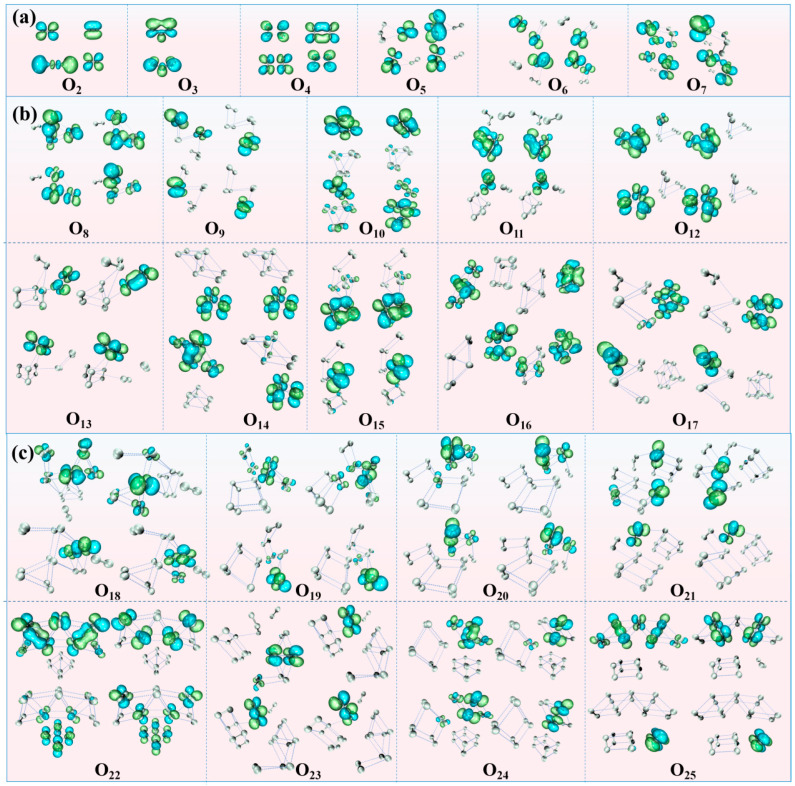
Diagrams of Frontier Molecular Orbitals. (Orbitals from left to right: α, β; from top to bottom: HOMO, LUMO. Isovalue = 0.02 a.u.; green/red = positive/negative orbital phase). (**a**) *n* = 2–7; (**b**) *n* = 8–17; (**c**) *n* = 18–25.

Figure 3a and Figure 4a illustrate the electrostatic potential distribution and frontier orbital diagrams for the smallest clusters in the series (*n* = 2–7). From an orbital perspective, the dense and continuous electron cloud bridges spanning oxygen atoms in O_2_ and O_3_ molecules indicate significant electron conjugation effects. The β-HOMO electron cloud of O_4_ spans four oxygen atoms, demonstrating effective electron cloud overlap and charge transfer between two O_2_ subunits. However, both α-LUMO and β-LUMO are localized within a single O_2_ subunit, indicating that while O_4_ exhibits electron delocalization and conjugation characteristics, its overall conjugation effect is weaker than that of O_2_ and O_3_. In the O_6_ cluster, the HOMO and LUMO are located on different O_2_ subunits without overlap, exhibiting certain chemical inertness. Analysis of the ESP diagrams of even-atom oxygen clusters reveals that both O_2_ and O_4_ display highly symmetric and uniform global electrostatic potential distributions, with no significant localized potential extrema on the surface. In the ground-state structure of the O_6_ cluster, O_2_ and O_4_ subunits are combined through weak electrostatic interactions at distances of 3.3–3.4 Å, maintaining a uniform electrostatic potential characteristic without distinct positive or negative charge centers. These electronic structural features, combined with the aforementioned thermodynamic stability analysis, corroborate the higher intrinsic stability of small even-atom clusters.

In contrast to even-atom clusters, odd-atom clusters exhibit significant positive and negative charge centers (orange-red regions represent negative charge, deep blue represents positive charge), with both centers located within the O_3_ subunit region. The V-shaped configuration of the O_3_ molecule exhibits a degree of symmetry, characterized by a positively charged central oxygen atom and negatively charged terminal oxygen atoms. The negative charge center lies along the line connecting the terminal oxygen atoms, while the positive and negative charge centers do not coincide, resulting in a large permanent dipole. This constitutes key electronic structural evidence for the odd–even oscillation effect in cluster dipole moments (as shown in Figure 2d dipole moment trend). The diagram of frontier molecular orbitals reveals that the HOMO and LUMO of O_3_ are degenerate, indicating its dual functionality as both a nucleophilic and electrophilic site, which accounts for its high reactivity. Coupled with its separated positive and negative charge centers and dipole, O_3_ exerts significant electronic polarization and induction effects on other oxygen atoms in odd-atom oxygen clusters. The frontier molecular orbitals diagram of O_5_ shows that the O_3_ subunit remains the electrophilic and nucleophilic center of the entire cluster. Due to the inductive effect of O_3_, strong electrostatic interactions occur between O_3_ and O_2_ subunits, resulting in uneven charge distribution on O_2_ (+0.030, −0.027) which is indicated by the red arrows in Figure 3a for O_5_. Similarly, O_7_ also displays distinct positive and negative charge centers, with the induction effect of O_3_ causing more uneven charges distribution on the O_2_ subunit (+0.113, −0.033) shown by arrows in Figure 3a for O_7_. The frontier molecular orbitals diagram demonstrates that the O_3_ subunit polarizes the O_2_ subunit, inducing degeneracy in its HOMO and LUMO and thereby reducing the energy gap, which significantly enhances the local reactivity. Thermodynamically, this active site facilitates cluster growth by incorporating new oxygen atoms, serving as an effective relaxation pathway to reduce the overall system energy and achieve stability.

Figure 3b and Figure 4b depict the electrostatic potential distributions and frontier orbitals for the intermediate-sized clusters (*n* = 8–17). Overall, the pattern of even–odd distribution differences aligns with that of small clusters (*n* = 2–7): even-atom clusters exhibit uniform electrostatic potential distributions, while odd-atom clusters still feature distinct positive and negative charge centers (deep blue and orange-red) located in the O_3_ subunits. Electrostatic potential interactions exist between all subunits, with particularly pronounced effects between O_3_ and other subunits. This further confirms the presence of weak interactions within the clusters, enabling oxygen atoms to form larger cluster structures.

Analysis of the frontier orbital distributions reveals partial overlap of the electron clouds on the O_6_ and O_8_ subunits in both odd and even clusters. This observation suggests a certain extent of electron delocalization within these subunits, implying that the O_6_ and O_8_ units inherently exhibit stability. Consequently, this intrinsic stability contributes to the enhanced overall stability of the larger cluster. In cluster O_9_, both the β-HOMO and LUMO are localized on the O_3_ subunit. A consistent localization pattern is observed for the α/β-LUMO in clusters O_11_, O_13_, O_15_, and O_17_. This persistent occupation of low-energy, unoccupied orbital space designates the O_3_ subunit as a strong electron acceptor with pronounced electron-withdrawing character. Consequently, it exerts a strong inductive effect on the electrons of neighboring O atoms, consistent with the electrostatic potential results showing significant electrostatic interactions between O_3_ and other subunits. For instance, cluster O_17_ comprises three subunits: O_3_, O_6_, and O_8_. O_8_ exhibits pronounced internal conjugation effects and is less influenced by O_3_. Conversely, O_6_, being closer to O_3_, experiences stronger induced dipole effects. Consequently, the bond distances between oxygen atoms of 1 and 3 (O1-O3) and atoms of 2 and 4 (O2-O4) within the O_6_ subunit elongate, reducing its stability.

Figure 3c and Figure 4c display the electrostatic potential distribution and frontier orbital diagrams for the largest clusters (*n* = 18–25). As oxygen clusters grow in size, both the dimensions and number of their constituent subunits increase. While weak electrostatic interactions remain the dominant inter-subunit force, the internal bonding within odd-atom clusters becomes weakened due to reduced electron overlap. This leads to the formation of loosely bound internal configurations, such as O_6_ + O_2_, O_8_ + O_2_, and O_8_ + O_2_ + O_2_. The large odd cluster O_19_ is composed of two O_8_ subunits and one O_3_ subunit. Notably, one O_8_ subunit is not a cuboid-like dense structure but instead forms an O_6_ + O_2_ structure. The HOMO and LUMO orbitals are localized precisely on this loose O_6_ + O_2_ structure and the O_3_ subunit, indicating that the induced effect from O_3_ reduces the local stability of the O_8_ subunit, thereby increasing the atomic activity within that subunit. The electrostatic potential analysis of O_19_ reveals that the O_3_ subunit, owing to its pronounced electron-withdrawing capacity, engages in substantial electrostatic interactions with the adjacent O13 atom. This interaction consequently attenuates the O-O bonds within the O_8_ subunit, thereby enhancing its reactivity. The O_25_ cluster consists of O_14_, O_8_, and O_3_ subunits. The LUMO orbitals are localized on the O_3_ subunit and the oxygen atoms in the O_14_ subunit adjacent to O_3_, while the HOMO orbitals are localized on the O_14_ subunit. The O_14_ subunit forms a network-like structure, with local electron delocalization occurring among the central oxygen atoms. This causes the edge oxygen atoms near O_3_, specifically O17 and O18, to be more susceptible to induction by the external O_3_ subunit. Therefore, the two oxygen atoms near O_3_ exhibit relatively higher activity and are prone to chemical reactions. Electrostatic potential analysis also shows that the O_3_ subunit in the O_25_ cluster, due to its strong electron-withdrawing ability, has significant electrostatic interaction with the nearby O18 atom.

A comprehensive analysis of the electrostatic potential distribution and frontier orbitals indicates that even-atom clusters exhibit stronger stability due to symmetrical structure and electron distribution. In contrast, odd-atom oxygen clusters experience internal electron density redistribution caused by the strong induction effect of the O_3_ subunit. When the cluster is small, the induction effect of O_3_ strongly polarizes the smaller subunits, making small clusters more inclined to capture external O_2_ molecules through electrostatic interactions, thus promoting cluster growth. As the cluster size increases, the size and number of internal subunits also increase, dispersing the induction effect of a single O_3_ subunit across multiple larger subunits. This leads to a reduction in the overall polarization strength on any single subunit. This “dilution” of the effect localizes the induction, thereby weakening a specific chemical bond and making dissociation (such as the dissociation of an oxygen radical) a more favorable reaction pathway than growth.

### 3.4. Molecular Dynamics Simulation of a Pure Oxygen System

Comprehensive analysis of the previous results indicates that the stability of oxygen clusters exhibits a pronounced odd–even oscillation trend as the number of oxygen atoms increases. The stability of odd-atom clusters is more closely related to their geometric and electronic structures, with the stability of larger odd-atom clusters diminishing. To further elucidate the growth kinetics mechanism of large-sized oxygen clusters, molecular dynamics simulations of a pure oxygen system were conducted at high temperature (1573 K) under different pressure conditions (3 atm and 6 atm). In our analysis, a cluster is counted if connectivity persists for ≥2 ps.

**Figure 5:**
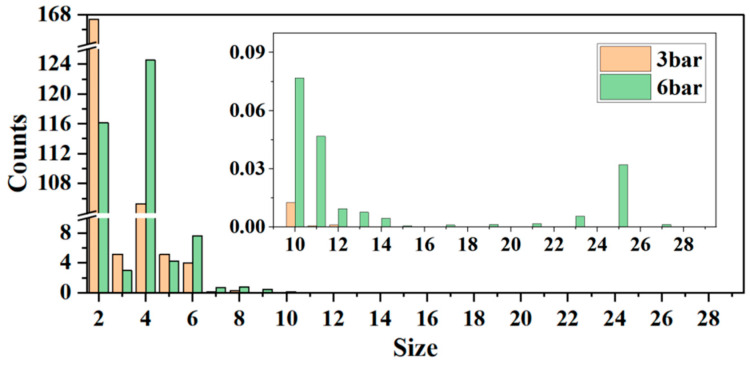
Effect of pressure on the distribution of oxygen clusters in a pure oxygen system.

Figure 5 compares the oxygen cluster population results at different sizes simulated under varying pressures in a pure oxygen system, revealing a clear trend: As the pressure increases from 3 atm to 6 atm, the maximum cluster size observed during the simulation period expands from O_13_ to O_31_. Concurrently, the population of clusters in the O_4_ and O_6_–O_14_ size ranges increases, while the number of O_2_ clusters decreases relatively. This change reveals the promotional effect of increased pressure on cluster nucleation and growth kinetics: Higher pressure increases the number density of O_2_ monomers and the effective collision frequency between molecules, significantly accelerating a series of elementary reaction rates—from initial nucleation (e.g., O_2_ + O_2_ → O_4_) to subsequent monomer-addition growth (e.g., O_n_ + O_2_ → O_n+2_). This leads to a reduction in O_2_ monomer numbers and an increase in large cluster numbers within the same time.

At pressures of 3 atm and 6 atm, the populations of O_2_ and O_4_ clusters are significantly greater than those of other sizes, indicating their superior kinetic stability. The average numbers of O_3_, O_5_, and O_6_ clusters are comparatively lower, each remaining under 10. Conversely, clusters larger than O_10_ exhibit a sharp decline, with average numbers below 0.1 during the simulation period, suggesting strong suppression of growth for large clusters. Within the interval spanning O_2_ to O_6_, even-atom oxygen clusters (O_2_, O_4_, O_6_) generally exhibited higher populations than adjacent odd-atom clusters (O_3_, O_5_), indicating thermodynamic and kinetic advantages for even clusters. This finding strongly aligns with the pronounced “odd–even oscillation” effect observed in stability analysis. However, this effect gradually diminishes with increasing cluster size, diverging from earlier structural analysis and thermodynamic calculations. This suggests that over larger size ranges, kinetic factors exert a significant influence and predominantly govern the behavior of cluster distribution, as the formation of larger clusters necessitates surmounting greater free energy barriers. The formation of large clusters relies on greater energy and more complex structural rearrangements, the likelihood of which diminishes substantially as cluster size increases. Therefore, from a kinetic standpoint, the formation of large clusters is markedly disfavored.

Analysis of the size distribution of clusters under 6 atm revealed a non-monotonic variation within the O_10_–O_28_ range: the population gradually decreased in the O_10_–O_15_ range, consistent with conventional nucleation kinetics; however, within the O_15_–O_25_ range, the population rebounded, reaching a local maximum at O_25_ before declining rapidly. This anomalous distribution is closely related to the structural evolution and stability of the clusters. Clusters comprising between 10 and 15 oxygen atoms predominantly include less stable O_4_ subunits. In contrast, as the cluster size expands to the range of 15 to 25 oxygen atoms, the clusters are mainly constituted by more stable subunits, specifically O_6_ and O_8_. Furthermore, the inductive effect of O_3_ is distributed among multiple subunits, which enhances the stability of the clusters. Consequently, the incidence of large clusters observed in the molecular dynamics simulations increases. The rapid decline after the O_25_ peak suggests a structural transition (e.g., release of radicals), reflecting a strong correlation between the stability of the constituent subunits and the structural evolution of the clusters.

To elucidate the microscopic mechanisms underlying these size distribution characteristics, this study further focuses on the kinetic evolution pathways from small clusters to larger ones. Under 6 atm, the system not only forms larger clusters (e.g., O_31_) but also exhibits structural transformation events triggered by the dissociation of large clusters, providing richer kinetic information for analyzing cluster formation and stability. Consequently, the following analysis will predominantly utilize the simulation trajectories obtained at 6 atm to perform a comprehensive investigation of the microscopic evolution mechanisms.

**Figure 6:**
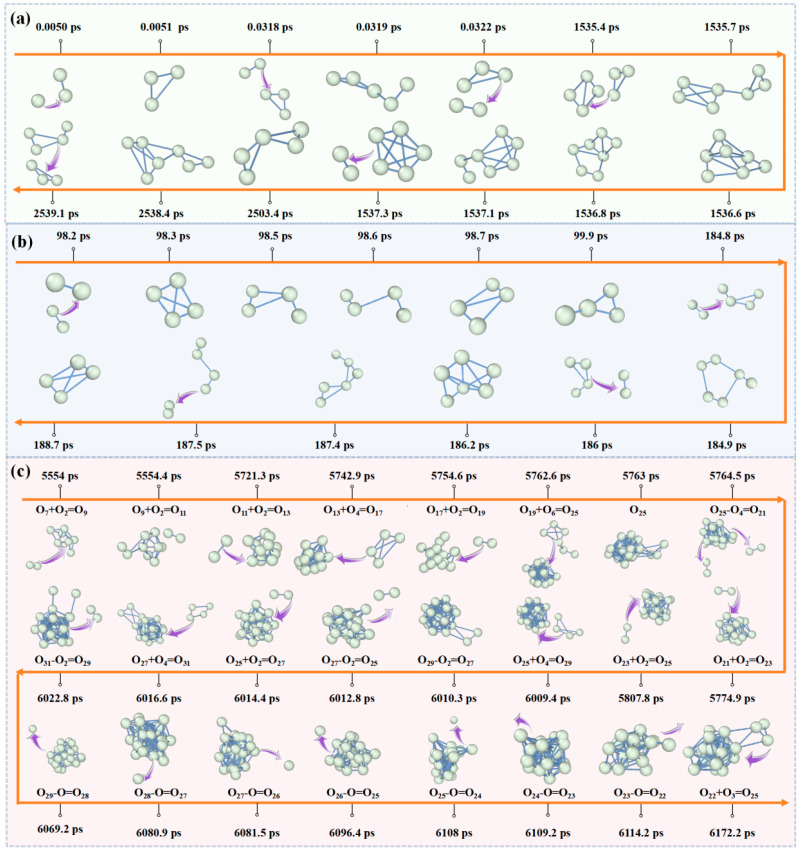
Evolution of cluster dynamics in a pure oxygen system. (**a**) Small odd-numbered clusters. (**b**) Small even-numbered clusters. (**c**) Large clusters and radical formation. Arrows indicate motion direction of little oxygen atom or molecule: binding (toward cluster) or dissociation (away from cluster).

Figure 6a illustrates the evolutionary process, demonstrating that in a pure oxygen system, the formation of O_3_ initiates from the combination reaction between O_2_ molecules and initially introduced oxygen radicals (O∙). The generated O_3_ tends to further interact with O_2_ molecules during subsequent motion, forming chain-like O_5_ clusters. However, due to their low stability, these chain-like O_5_ clusters often rapidly dissociate, regenerating O_2_ and O_3_, thereby facilitating oxygen atom exchange. Kinetic simulation trajectories indicate that O_3_ can also directly combine with O_4_ to form O_7_ clusters, whose structures exhibit dynamic structural characteristics. At 1536.8 ps in the kinetic simulation, a cyclic O_5_ structure is observed to combine with O_2_; as vibrations continue, their interaction gradually weakens, and they dissociate at 1537.3 ps, regenerating cyclic O_5_ and O_2_. Compared to chain-like O_5_, cyclic O_5_ exhibits higher kinetic stability due to electron delocalization effects, resulting in a longer residence time in the system (approximately 2 ns). After 2503.4 ps in the kinetic simulation, the cyclic O_5_ undergoes ring-opening transformation, forming a chain-like configuration and combining with O_2_ to generate O_7_, which subsequently rapidly dissociates into O_4_ and O_3_.

During the evolution of oxygen clusters (Figure 6b), O_4_ not only engages in reversible combination-dissociation behavior with O_3_ but also interacts with O_2_. The O_2_-O_4_-O_6_ evolutionary pathway reveals a collision-driven reversible reaction mechanism. Initially, two O_2_ molecules combine through collision to form an O_4_ cluster. This O_4_ cluster can further collide with free O_2_ molecules in the system, generating O_6_ clusters via the reversible reaction O_4_ + O_2_ ⇌ O_6_. However, O_6_ clusters are metastable with extremely short lifetimes, rapidly dissociating into O_2_ and O_4_. Through continuous reversible combination and dissociation with O_2_ molecules, O_4_ clusters not only achieve dynamic equilibrium between O_4_ and O_6_ but also promote oxygen atom exchange among different clusters, providing microscopic pathways for oxygen atom migration.

Molecular dynamics trajectory analysis of larger oxygen cluster evolutionary pathways (Figure 6c) indicates that their growth mechanism primarily relies on reversible adsorption and dissociation with smaller subunits (e.g., O_2_, O_4_, O_6_). The initial O_9_ cluster gradually increases in size through continuous reactions with such subunits. During the time interval from 5762.6 to 5764.5 ps, O_19_ is observed to combine with O_6_ to generate O_25_, which rapidly dissociates, releasing two O_2_ molecules and forming an O_21_ cluster. This phenomenon suggests that, compared to larger subunits (e.g., O_6_), the combination of smaller subunits (e.g., O_2_) with clusters is kinetically more favorable.

As the size continues to increase, the cluster structure begins to undergo significant changes. At 6016.6 ps, O_27_ combines with O_4_ to form O_31_, which dissociates after 6.2 ps, releasing one O_2_ to become O_29_. At this point, the large odd-atom cluster O_29_ initiates O-O bond dissociation, releasing oxygen radicals and diminishing to O_22_ within 45 ps (at 6114.2 ps). The detection of radical release from the sizable odd-atom cluster O_29_ provides corroborative evidence supporting previous theoretical analyses: Structural analysis identifies relaxation features within the large cluster—including expanded interatomic distances both within and between subunits, as well as altered dihedral angles—providing a structural basis for its instability. The decrease in the HOMO-LUMO gap and the increased local atomic activity within large subunits (e.g., O_8_, O_14_), as revealed by electronic structure analysis, are the electronic structural origins of the enhanced reactivity. The release of free radicals is a necessary outcome of the kinetic instability caused by the aforementioned structural relaxation and increased local atomic activity. At 6172.2 ps, the cluster subsequently re-enters a phase of aggregated growth with small subunits such as O_2_. This entire process reveals a dynamic growth pattern for oxygen clusters: their size does not increase in a strictly monotonic manner; rather, it undergoes dynamic modulation through reversible interactions among subunits and internal induced dipole effect, which initiate bond cleavage and the release of oxygen radicals, thereby promoting atomic exchange. This “growth-dissociation” dynamic cycle demonstrates that oxygen cluster evolution is jointly governed by thermodynamic stability and kinetic activity: elevated kinetic activity enables atomic exchange and structural rearrangement, while thermodynamic stability ultimately guides the system toward forming more structurally stable cluster morphologies, establishing a dynamic equilibrium between these two processes.

### 3.5. MD Simulation of the Titanium Tetrachloride-Oxygen System

To investigate the formation of oxygen clusters and radical release mechanisms under realistic combustion conditions, molecular dynamics simulations were further conducted in the titanium tetrachloride oxidation system (temperature: 1573 K, pressure: 6 atm).

**Figure 7:**
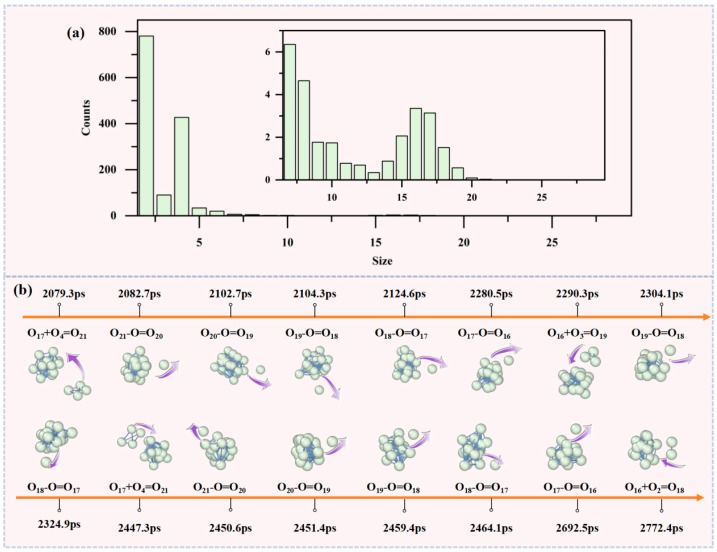
Evolution results of oxygen clusters in the titanium tetrachloride oxidation system. (**a**) Variation in the number of oxygen clusters with cluster size. (**b**) Process of cluster growth and free radical release. Arrows indicate motion direction of little oxygen atom or molecule: binding (toward cluster) or dissociation (away from cluster).

Figure 7a indicates the variation in the number of oxygen clusters with cluster size, demonstrating that O_2_ and O_4_ are the most abundant species, consistent with results from pure oxygen systems. This indicates that O_2_ and O_4_ possess optimal kinetic stability in both oxygen and oxygen-rich oxidation environments. As the cluster size *n* increases further, the population of large clusters rapidly decreases; however, after *n* > 13, the population rises again, forming a peak in the O_15_–O_17_ range, with O_16_ exhibiting the highest population and greatest stability. When *n* > 16, the number of clusters decreases again. Although the maximum cluster size extends to O_28_, clusters in the O_22_–O_28_ range are very scarce. Larger clusters quickly enter metastable states and rapidly release free radicals. This evolutionary pattern is basically consistent with that in pure oxygen environments. The stability of oxygen clusters does not change monotonically with size but shows a clear nonlinear relationship. Stable O_6_ and O_8_ subunits also serve as characteristic structural units, enhancing the stability of large clusters larger than O_13_. This further confirms that O_6_ and O_8_ subunits play a key stabilizing role in large clusters under complex reaction conditions.

Similar to the pure oxygen system, Figure 7b shows the process of molecular dynamics simulations of the titanium tetrachloride oxidation system at 6 atm, revealing that oxygen clusters continue to grow through the dynamic association and dissociation of smaller O*_n_* (*n* = 2–6) clusters with existing clusters. Upon reaching O_21_, the oxygen clusters commence a rapid and continuous liberation of free radicals. As the clusters reduce to O_16_, they subsequently merge with smaller clusters, including O_2_ and O_4_, to facilitate further growth, entering a cycle of releasing free radicals (O∙) and merging with O_n_ clusters. As demonstrated in Section 3.3, high pressure (6 atm) in the pure oxygen system drives rapid cluster growth up to O_29_ before any radical release occurs. However, in the TiCl_4_ oxidation system at the same pressure, radical release occurs at a smaller cluster size of O_21_, indicating that TiCl_4_ addition has a dual effect on oxygen cluster evolution. Firstly, the steric hindrance effect of TiCl_4_ molecules in physical space impedes effective contact and collision between oxygen molecules (O_2_) and existing oxygen clusters, thereby suppressing the formation of large clusters and shifting the maximum cluster size distribution towards smaller dimensions. More significantly, the chemical catalysis and energy supply role of TiCl_4_ profoundly participates in the oxidation reaction, releasing substantial energy during the process. This high-density energy is preferentially absorbed by adjacent, unstable oxygen clusters, particularly those containing strongly inductive O_3_ subunits in odd-atom clusters, significantly accelerating the desorption rate of free radicals (O·). This suggests that TiCl_4_ may alter the pathway of free radical formation, shifting the system from a physical transformation pathway dependent on large clusters to a chemical pathway reliant on rapid free radical generation from smaller clusters, thereby enhancing the overall reactivity of the system. This observation underscores the critical role of kinetic factors in influencing free radical release.

In both the pure oxygen system and the high-temperature TiCl_4_ oxidation system, the evolution pathways of oxygen clusters and the process of radical release exhibit a high degree of consistency. This finding indicates that oxygen radicals are primarily generated through the intrinsic cluster evolution mechanism of oxygen molecules themselves, rather than necessitating direct participation of TiCl_4_ molecules. The role of TiCl_4_ appears to be more inclined towards modulating the thermodynamic equilibrium and kinetic rates of oxygen cluster formation, rather than constructing an entirely new reaction pathway. This oxygen cluster evolution mechanism, serving as a universal channel for radical generation, not only deepens the understanding of the titanium tetrachloride oxidation system but also provides a novel microscopic perspective and theoretical model for interpreting a broader range of catalytic oxidation and combustion phenomena.

## 4. Conclusions

In summary, through multi-scale computational simulations, we have revealed a previously unrecognized yet fundamental pathway for radical generation in high-temperature oxidation: the formation, evolution, and collapse of transient oxygen clusters. Contrary to the conventional view of oxygen as discrete O_2_ molecules, we demonstrate that oxygen at high temperatures exists as dynamic van der Waals aggregates whose stability follows a distinct odd–even oscillation pattern. This oscillation is governed by the interplay between the geometric stability of even-numbered subunits (O_2_, O_4_, O_6_, O_8_) and the electronic activity of O_3_ units, with the latter serving as the key driver of radical release.

Mechanistically, cluster evolution proceeds via a reversible growth–dissociation pathway, wherein radical release is not determined by a specific cluster size but is regulated by both system pressure and chemical environment. The persistence of this “oxygen clustering–radical release” mechanism in both pure O_2_ and TiCl_4_-containing systems underscores its universality, with TiCl_4_ primarily modulating aggregation kinetics rather than altering the fundamental pathway.

These findings shift the paradigm for understanding high-temperature oxidation from a molecular to a supramolecular perspective, establishing oxygen clustering as a spontaneous, intrinsic source of radicals. The proposed model provides a new theoretical framework for explaining non-equilibrium radical generation and offers mechanistic insights relevant to materials synthesis, corrosion, combustion chemistry, and other high-temperature processes where radical-mediated reactions play a decisive role.

## Data Availability

The original contributions presented in this study are included in the article/Supplementary Material. Further inquiries can be directed to the corresponding authors.

## Supplementary Materials

The following supporting information can be downloaded at: https://www.mdpi.com/article/10.3390/ma19061048/s1. Figure S1: (a) Loss curves of energy and force. (b) Scatter plot of the predicted values (DP) and true values (DFT). Table S1: The dihedral angles of clusters and their characteristic substructure units. Figure S2: evolution of the oxygen cluster size distribution (a) 3atm (b) 6atm. Table S2: The HOMO and LUMO energy values of clusters/eV. Table S3: The lowest energy of key species/eV.

## References

[1] Totton T.S., Shirley R., Kraft M. (2011). First-principles thermochemistry for the combustion of TiCl_4_ in a methane flame. Proc. Combust. Inst..

[2] West R.H., Shirley R.A., Kraft M., Goldsmith C.F., Green W.H. (2009). A detailed kinetic model for combustion synthesis of titania from TiCl_4_. Combust. Flame.

[3] Shirley R., Liu Y., Totton T.S., West R.H., Kraft M. (2009). First-Principles Thermochemistry for the Combustion of a TiCl_4_ and AlCl_3_ Mixture. J. Phys. Chem. A.

[4] Zhao S., Yu Y., Song Y., Jia Y., Wang Q. (2025). Skeletal oxidation mechanism for n-Dodecane in gas turbine combustor simulations. Chem. Eng. J..

[5] Zhou Z., Yi Q., Wang R., Wang G., Ma C. (2020). Numerical Investigation on Coal Combustion in Ultralow CO_2_ Blast Furnace: Effect of Oxygen Temperature. Processes.

[6] Li J., Chen Z., Zhang X., Qiao Y., Yuan Z., Li Z. (2023). Thermal conversion, kinetics, thermodynamics and empirical optimization of combustion performance of coal gasification fine ash in oxygen-enriched atmosphere. Fuel.

[7] Grundland I. (1953). Etude au Spectrometre de Masse de Polymeres de Loxygene Presents dans Loxygene Ozonise (O_3_ et O_4_). Comptes Rendus Hebd. Seances L’Acad. Sci..

[8] Lipikhin N.P. (1975). The Oxygen Dimers, Clusters, and Cluster Ions in the Gas Phase. Russ. Chem. Rev..

[9] Cabria I., López M.J., March N.H., Alonso J.A. (2013). Evolution of the atomic structure and the magnetism of small oxygen clusters. Comput. Theor. Chem..

[10] Hernández-Lamoneda R., Pérez-Ríos J., Carmona-Novillo E., Bartolomei M., Campos-Martínez J., Hernández M.I. (2012). Properties of the molecular oxygen trimer from pairwise additive interactions. Chem. Phys..

[11] Calvo F., Torchet G. (2007). Onset of crystalline order in oxygen clusters. J. Cryst. Growth.

[12] Santoro M., Dziubek K., Scelta D., Morana M., Gorelli F.A., Bini R., Hanfland M., Rouquette J., di Renzo F., Haines J. (2019). Dense, Subnano Phase of Clustered O_2_. J. Phys. Chem. C.

[13] Gadzhiev O.B., Ignatov S.K., Kulikov M.Y., Feigin A.M., Razuvaev A.G., Sennikov P.G., Schrems O. (2013). Structure, Energy, and Vibrational Frequencies of Oxygen Allotropes O_n_ (n ≤ 6) in the Covalently Bound and van der Waals Forms: Ab Initio Study at the CCSD(T) Level. J. Chem. Theory Comput..

[14] Forte G., Angilella G.G.N., March N.H., Pucci R. (2013). The nuclear structure and related properties of some low-lying isomers of free-space O_n_ clusters (*n* = 6, 8, 12). Phys. Lett. A.

[15] Conway D.C. (1969). Possible O_6_^+^ Structures Obtained by a Semiempirical SCF–MO Method. J. Chem. Phys..

[16] Conway D.C., Janik G.S. (1970). Determination of the Bond Energies for the Series O_2_–O_2_^+^ through O_2_–O_10_^+^. J. Chem. Phys..

[17] Conway D.C. (1969). Geometries of O_4_^+^, O_4_^−^, and N_4_^+^ by an Approximate SCF–MO Theory Which Considers Intermolecular Differential Overlap. J. Chem. Phys..

[18] Conway D.C. (1970). Possible O_8_^+^–O_12_^+^ Structures Obtained by Use of Classical Electrostatic Theory. J. Chem. Phys..

[19] Li J., Huang H., Huhetaoli, Osaka Y., Bai Y., Kobayashi N., Chen Y. (2017). Combustion and Heat Release Characteristics of Biogas under Hydrogen- and Oxygen-Enriched Condition. Energies.

[20] Yamamoto T., Tsuboi T., Iwama Y., Tanaka R. (2016). Combustion and Reformulation Enhancement Characteristics of Plasma-Assisted Spray Combustion by Microwave-Induced Non-Equilibrium Plasma. Energy Fuels.

[21] Sun J., Ravelid J., Bao Y., Nilsson S., Konnov A.A., Ehn A. (2024). Dynamics of atomic oxygen production in an NH_3_/air flame assisted by a nanosecond pulsed plasma discharge. Proc. Combust. Inst..

[22] Song G., Bozzelli J.W. (2019). Reaction pathways, kinetics and thermochemistry of the chemically-activated and stabilized primary methyl radical of methyl ethyl sulfide, CH_3_CH_2_SCH_2_•, with 3O_2_ to CH_2_CH_3_SCH_2_OO•. Combust. Flame.

[23] Zhang Y., Zhang W., Yu B., Li X., Zhang L., Zhao Y., Sun S. (2024). Experimental and kinetic modeling study on laminar flame speeds and emission characteristics of oxy-ammonia premixed flames. Int. J. Hydrogen Energy.

[24] Chen L., Qi X., Tang J., Xin H., Liang Z. (2021). Reaction pathways and cyclic chain model of free radicals during coal spontaneous combustion. Fuel.

[25] Bazarkina E.F., Bauters S., Watier Y., Weiss S., Butorin S.M., Kvashnina K.O. (2025). Exploring cluster formation in uranium oxidation using high resolution X-ray spectroscopy at elevated temperatures. Commun. Mater..

[26] Liu X., Wang R., Huang T., Geng X., Ding X., Duan Y., Zhao S. (2023). Insights into the HCl formation and volatilization mechanism from organochlorine in coal: A DFT study. Fuel.

[27] Ding Z., Selloni A. (2023). Modeling the aqueous interface of amorphous TiO_2_ using deep potential molecular dynamics. J. Chem. Phys..

[28] Liu H., Ma Y., Mu D., Shang F., Lv M., Shan J., Yin S., Liu J. (2024). Detailed Microscopic Reaction Mechanism during the Combustion Process of HAN-Based Propellant. J. Phys. Chem. A.

[29] An Q., Basem A., Alizadeh A., Al-Rubaye A.H., Jasim D.J., Tang M., Salahshour S., Sabetvand R. (2024). The effect of initial temperature and oxygen ratio on air-methane catalytic combustion in a helical microchannel using molecular dynamics approach. Case Stud. Therm. Eng..

[30] Liu B., Chen X., Zhang E., Zhou J., Wu H., Chen Y., Yang B., Xu B., Jiang W. (2024). Formation mechanism and luminescence properties of silicon carbide nanowires with core-shell structure. Ceram. Int..

[31] Meunier M. (2008). Guest Editorial. Mol. Simul..

[32] Zhao Y., Truhlar D.G. (2008). Density Functionals with Broad Applicability in Chemistry. Acc. Chem. Res..

[33] Bondi A. (1964). van der Waals Volumes and Radii. J. Phys. Chem..

[34] Chan H., Narayanan B., Cherukara M.J., Sen F.G., Sasikumar K., Gray S.K., Chan M.K.Y., Sankaranarayanan S.K.R.S. (2019). Machine Learning Classical Interatomic Potentials for Molecular Dynamics from First-Principles Training Data (Review). J. Phys. Chem. C.

[35] Li D., Li L., Lu P., Zhou J., Chen Y., Sheng Z., Li L., Chen X., Liu D. (2026). Unveiling the High-Temperature oxidation mechanism of TiCl4 via deep potential molecular dynamics toward TiO_2_ synthesis. Chem. Eng. Sci..

[36] Chen X., Chen Y., Zhou J. (2026). Short bond evaluation method for rapidly assessing the generalization ability of deep neural network potential function models and its effectiveness verification. Npj Comput. Mater..

[37] Wang H., Zhang L., Han J., E W. (2018). Deepmd-kit: A deep learning package for many-body potential energy representation and molecular dynamics. Comput. Phys. Commun..

[38] Zeng Q., Chen B., Yu X., Zhang S., Kang D., Wang H., Dai J. (2022). Towards large-scale and spatio-temporally resolved diagnosis of electronic density of states by deep learning. Phys. Rev. B.

[39] Nosé S. (2014). A unified formation of the constant temperature molecular dynamics methods. J. Chem. Phys..

[40] Rao B.K., Jena P. (1999). Evolution of the electronic structure and properties of neutral and charged aluminum clusters: A comprehensive analysis. J. Chem. Phys..

[41] Guo L., Yang Y. (2013). Theoretical investigation of molecular hydrogen adsorption and dissociation on Al_n_V (*n* = 1–13) clusters. Int. J. Hydrogen Energy.

