# Supplementary material for: Evolution of High-Temperature Oxygen Clusters and Radical Release: A Molecular Dynamics Study in Pure Oxygen and Titanium Tetrachloride Oxidation Environments
Source: Materials (Basel). 2026 Mar 10;19(6):1048. doi: 10.3390/ma19061048 (PMC13028297; doi:10.3390/ma19061048)
Supplement: Supplementary file 1 [file materials-19-01048-s001.zip › materials-4155324-supplementary.pdf]

Supplement S1
Method of Deep Potential Molecular Dynamics Calculation (DPMD)

A dataset containing approximately 120,000 data points obtained from first-principles molecular dynamics simulations (including structures of O_2 clusters) at 973-2273K was used for deep learning training[34], of which 80% was randomly selected to form the training set and 20% was used as the test set. The training parameters are as follows: The smooth version of Deep MD-kit[37] 2.2.8 is used, and the descriptor keyword "type" is "se_e2_a". According to the system characteristics, rcut_smth was set to the default value for expressing system interactions and r_cut was set to 6Å. The embedding neural network was set to (20, 40, 80), and the deep neural network was configured to (200, 200, 200). The learning rate for the training process was initialized at 0.001 and gradually decreases to 3.51×10^{-8} by the end. The decay type was specified as exponential (exp), and the learning rate decay_steps parameter was adjusted to 7,600 . The weight coefficients $P_{\varepsilon}^{\text{start}}$ $P_{\text{f}}^{\text{start}}$ $P_{\varepsilon}^{\text{limit}}$ $P_{\text{f}}^{\text{limit}}$, corresponding to the initial and final times are assigned values of 0.02, 1000, 1, and 1 , respectively, as only energy and force are required to be accounted for when constructing the loss function. Additionally, the maximum number of training steps was set to 1,520,000. The initial configuration of the $TiCl_4$ and O_2 reaction system was established using a trained potential function with the help of the Amorphous Cell module of the Materials Studio package.

During the training process, the generalization ability of the model was be effectively evaluated by observing the loss curves of both the training and validation sets[38]. Fig.1(a) presents the training loss and validation loss curves for both energy and force, which reveal that the root mean square error (RMSE) of the force and energy is approximately 10^{-1}eV/Å, while the RMSE for the energy per atom is approximately 10^{-3} eV. These prediction performances satisfied the accuracy requirements of the system, implying that the model is suitable for subsequent simulation tasks.

After training was completed, the fitting performance of the model was observed by comparing the scatter plots of the predicted values (Deep Potential, DP) and the real values (density functional theory, DFT) in the validation set. The abscissa in Fig.1(b) corresponds to the DFT-derived data, whereas the ordinate represents the DP-computed outputs. The mean absolute error (MAE) of the atomic energy, as determined through validation-set calculations, was 2.56×10^{-3} eV. The force MAE in the three coordinate directions was approximately 0.12 eV/Å. Therefore, the close agreement between the model predictions and true values in the validation set indicates that the model is suitable for the titanium tetrachloride oxidation reaction

system.

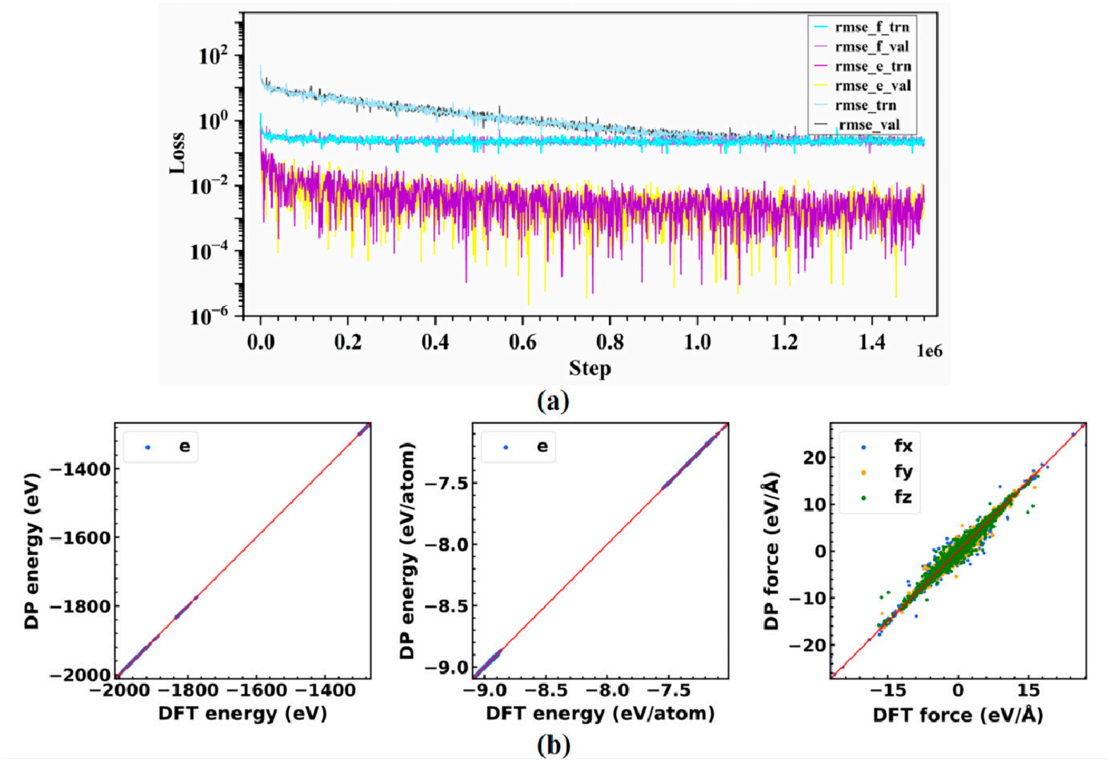

FIG. S1 (a) Loss curves of energy and force, (b) Scatter plot of the predicted values (DP) and true values (DFT).

Supplement S2

Table S1 The dihedral angles of clusters and their characteristic substructure units

	Clusters	Dihedral	Coordination	comment
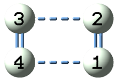	O_4	179.869	1-2-3-4	
		171.173	2-4-6-5	
		169.852	4-2-5-6	
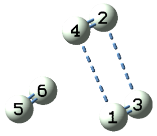	O_6	170.53	1-3-5-6	
		174.393	1-3-4-2	For subunit of O_4
		170.764	3-1-6-5	
		-176.837	4-3-5-2	
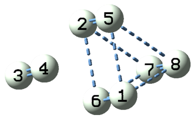	O_8	-177.543	1-6-4-3	
		-179.695	1-6-5-2	For subunit of O_6
		-179.77	8-7-5-2	
		179.971	7-8-5-6	
		179.769	8-7-14-13	For subunit of O_8
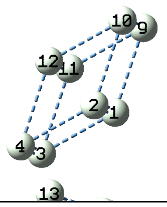	O_{16}	-179.638	15-16-13-14	
		179.889	6-5-16-15	
		-179.845	3-4-11-12	For subunit of O_8

		-179.843	3-4-1-2	
		179.948	9-10-1-2	
		-179.984	9-10-11-12	
		-167.943	18-17-20-21	
		159.664	22-23-12-11	
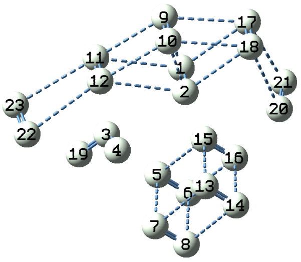	O_{23}	-178.339	18-17-10-9	
				For subunit of O_8
		-179.693	18-17-2-1	

Supplement S3

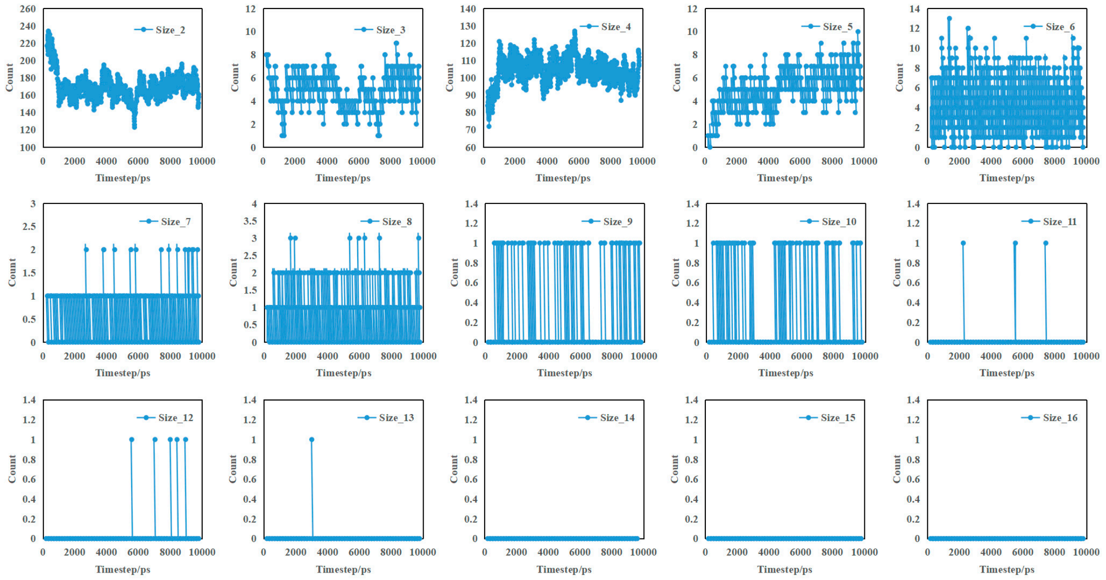

(a)

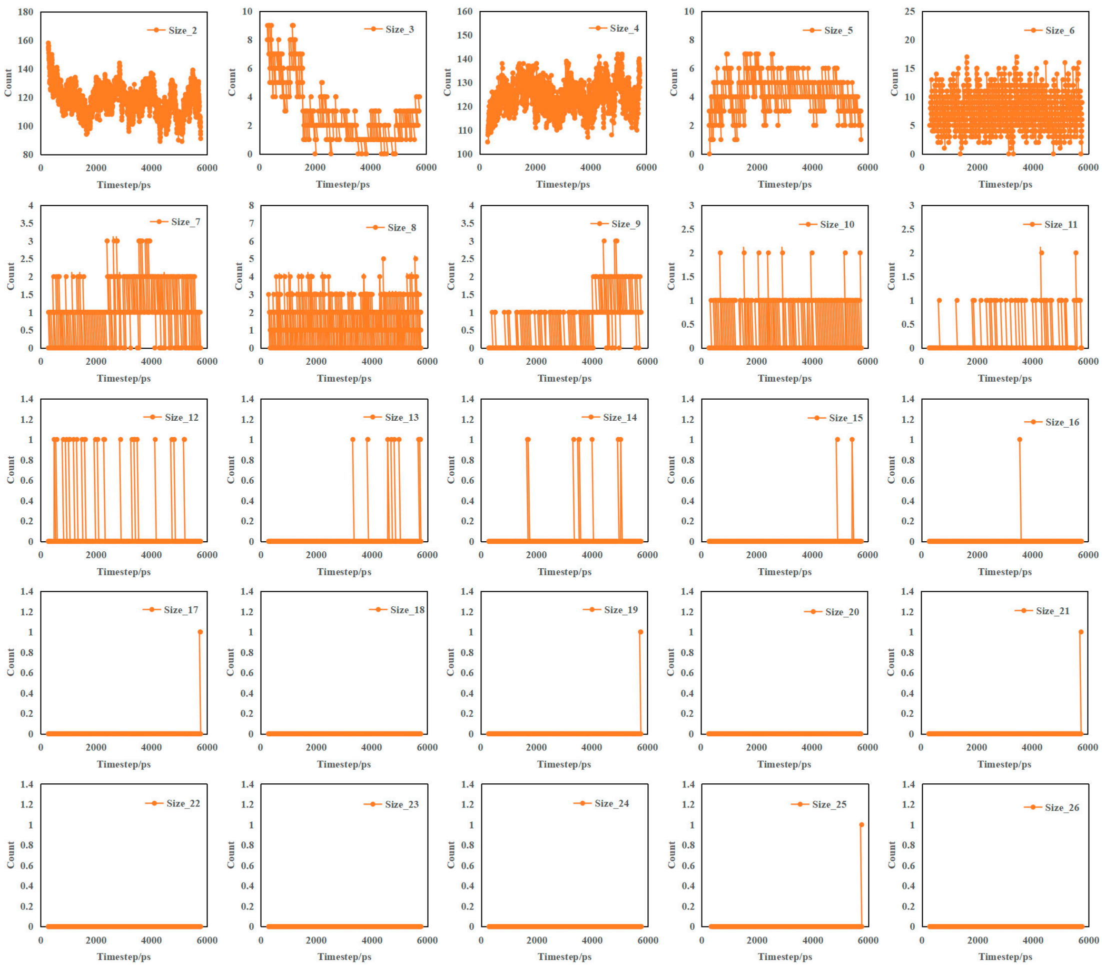

(b)

FIG. S2 evolution of the oxygen cluster size distribution (a) 3atm (b) 6atm

Supplement S4

Table S2 The HOMO and LUMO energy values of clusters/eV

Cluster Size	HOMO-α	LUMO-α	gap-α	HOMO-β	LUMO-β	gap-β	HOMO-LUMO Gap
2	-0.27043	0.07958	0.35001	-0.43754	-0.15978	0.27776	0.27776
3	-0.3028	-0.22755	0.07525	-	-	-	0.07525
4	-0.25162	-0.18761	0.06401	-0.2545	-0.18568	0.06882	0.06401
5	-0.27152	-0.22606	0.04546	-0.3017	-0.22608	0.07562	0.04546
6	-0.26967	-0.16181	0.10786	-0.27189	-0.16475	0.10714	0.10714
7	-0.265	-0.21016	0.05484	-0.26753	-0.21524	0.05229	0.05229
8	-0.2535	-0.18494	0.06856	-0.254	-0.18567	0.06833	0.06833
9	-0.27029	-0.22768	0.04261	-0.27018	-0.22782	0.04236	0.04236
10	-0.25159	-0.18773	0.06386	-0.25423	-0.18757	0.06666	0.06386

11	-0.2617	-0.22008	0.04162	-0.26182	-0.22053	0.04129	0.04129
12	-0.25904	-0.18686	0.07218	-0.25942	-0.18686	0.07256	0.07218
13	-0.2526	-0.22384	0.02876	-0.25593	-0.22384	0.03209	0.02876
14	-0.25017	-0.18789	0.06228	-0.2518	-0.18789	0.06391	0.06228
15	-0.26227	-0.21838	0.04389	-0.26278	-0.21845	0.04433	0.04389
16	-0.25088	-0.18895	0.06193	-0.25171	-0.189	0.06271	0.06193
17	-0.26041	-0.22292	0.03749	-0.2608	-0.22285	0.03795	0.03749
18	-0.24965	-0.19018	0.05947	-0.25163	-0.18672	0.06491	0.05947
19	-0.25713	-0.2164	0.04073	-0.25749	-0.21647	0.04102	0.04073
20	-0.25418	-0.18674	0.06744	-0.25469	-0.18635	0.06834	0.06744
21	-0.26631	-0.22856	0.03775	-0.26291	-0.22855	0.03436	0.03436
22	-0.24834	-0.19063	0.05771	-0.24711	-0.19116	0.05595	0.05595
23	-0.25273	-0.21866	0.03407	-0.25206	-0.21836	0.03370	0.03370
24	-0.25027	-0.18205	0.06822	-0.25081	-0.18524	0.06557	0.06557
25	-0.24825	-0.22415	0.02410	-0.24799	-0.22383	0.02416	0.02410

Supplement S5

Table S3 The lowest energy of key species/eV

Cluster Size	multiplicity=1	multiplicity=3	The lowest energy
2	-150.29	-150.35	-150.35
3	-225.46	-225.41	-225.46
4	-300.52	-300.68	-300.68
5	-375.76	-375.81	-375.81
6	-450.99	-451.06	-451.06
7	-526.09	-526.15	-526.15
8	-601.33	-601.40	-601.40
9	-676.50	-676.52	-676.52
10	-751.70	-751.72	-751.72
11	-826.82	-826.85	-826.85
12	-902.04	-902.09	-902.09
13	-977.17	-977.19	-977.19
14	-1052.38	-1052.43	-1052.43
15	-1127.48	-1127.56	-1127.56
16	-1202.71	-1202.78	-1202.78
17	-1277.82	-1277.91	-1277.91

18	-1353.04	-1353.15	-1353.15
19	-1428.19	-1428.29	-1428.29
20	-1503.43	-1503.53	-1503.53
21	-1578.52	-1578.66	-1578.66
22	-1653.73	-1653.83	-1653.83
23	-1728.86	-1729.00	-1729.00
24	-1804.08	-1804.24	-1804.24
25	-1879.16	-1879.34	-1879.34

[34]Chan, H.; Narayanan, B.; Cherukara, M.J.; Sen, F.G.; Sasikumar, K.; Gray, S.K.; Chan, M.K.Y.; Sankaranarayanan, S.K.R.S. Machine Learning Classical Interatomic Potentials for Molecular Dynamics from First-Principles Training Data (Review). J. Phys. Chem. C. 2019, 123, 6941–6957. https://doi.org/10.1021/acs.jpcc.8b09917.

[37] H.W.H.S. Wang, L.Z.L.S. Zhang, J.H.J.S. Han, W.E.W.S. E, Deepmd-kit: a deep learning package for many-body potential energy representation and molecular dynamics., Comput. Phys. Commun. 228 (2018) 178-184. https://doi.org/10.1016/j.cpc.2018.03.016.

[38] B.C.X.Y. Qiyu Zeng, Towards large-scale and spatio-temporally resolved diagnosis of electronic density of states by deep learning, Phys. Rev. B. 105 (2022) 174109. https://doi.org/10.48550.
